# Effects of Chronic Oral Probiotic Treatment in Paclitaxel-Induced Neuropathic Pain

**DOI:** 10.3390/biomedicines9040346

**Published:** 2021-03-30

**Authors:** Mariarosaria Cuozzo, Vanessa Castelli, Carmen Avagliano, Annamaria Cimini, Michele d’Angelo, Claudia Cristiano, Roberto Russo

**Affiliations:** 1 Department of Pharmacy, University of Naples Federico II, 80131 Napoli, Italy; mariarosaria.cuozzo@unina.it (M.C.); carmen.avagliano@gmail.com (C.A.); roberto.russo@unina.it (R.R.); 2 Department of Life, Health and Environmental Sciences, University of L’Aquila, 67100 L’Aquila, Italy; vanessa.castelli@univaq.it (V.C.); annamaria.cimini@univaq.it (A.C.); 3 Sbarro Institute for Cancer Research and Molecular Medicine and Center for Biotechnology, Temple University, Philadelphia, PA 19122, USA; 4 Task Force on Microbiome Studies, University of Naples “Federico II”, 80131 Naples, Italy

**Keywords:** chemotherapeutics, neuropathic pain, SLAB51, inflammation, gut integrity, spinal cord

## Abstract

Chemotherapy-induced peripheral neuropathy (CIPN) represents one of the most prevalent and potentially disabling side effects due to the use of anticancer drugs, one of the primary neuropathies detected is peripheral neuropathy induced by administration of taxanes, including paclitaxel. It has been demonstrated that gut microbiota is crucial for the therapeutic effect of chemotherapeutic drugs for inhibiting tumor growth and contributed to the pathogenesis of the CIPN. The use of nutraceuticals has receiving growing attention from the research community due to their phytochemical, biological, and pharmacological properties. It has been demonstrated that probiotic formulations may both reduce inflammation and modulate the expression of pain receptors. Our studies tested the efficacy of a probiotic formulation, SLAB51, in preventing paclitaxel-induced neuropathy. Interestingly, our probiotic formulation was able to keep the gut integrity, preserving its functionality, in CIPN-mice, moreover, it prevented the mechanical and cold hypersensitivity induced in paclitaxel-mice. Additionally, ex-vivo analysis showed that in CIPN-mice the pro-biotic treatment increased the expression of opioid and cannabinoid receptors in spinal cord, it prevented in the reduction in nerve fiber damage in the paws and modulated the serum proinflammatory cytokines concentration. On basis of these data, the use of this specific probiotic formulation may represent a valid adjuvant agent to paclitaxel, useful and not toxic for long-lasting therapies.

## 1. Introduction

Chemotherapeutics are commonly used for tumor therapy, but they also trigger numerous detrimental side effects. Chemotherapy-induced peripheral neuropathy (CIPN) represents one of the prevalent and potentially disabling side effects due to the use of anticancer drugs [1]. The most frequent signs are tingling, numbness, or pain in the extremities [1,2]. In a recent meta-analysis including 4179 patients upon chemotherapies, the incidence of CIPN was about 70% within the first month, 60.0% at three months, and 30.0% at six months of therapy [3]. CIPN has numerous clinical implications that significantly affect the patients’ quality of life, leading to the interruption of therapy, which could eventually impact overall survival [4]. One of the primary neuropathy detected is peripheral neuropathy induced by taxane administration, including paclitaxel (PTX) [5]. PTX is widely applied for the treatment of feminine cancers, gastrointestinal and lung cancers [6,7,8].

Despite the intensive research focused on treating or preventing CIPN [9], efficacious pharmacological agents are lacking. PTX-induced neuropathic pain represents sensory neuropathy. In particular, low doses of PTX triggers hypersensitivity to pain, including allodynia and hyperalgesia, while high doses led to axonal degeneration to the peripheral nerves [5]. Neurotoxicity induced by PTX is ascribed to altered microtubule structure inducing enhanced microtubule stability and increased acetylated α-tubulin and consequently neuropathic pain, parallel with the activation of IL8 signaling in DRG neurons in culture [10].

It has been demonstrated that the gut microbiota is crucial for the therapeutic effect of chemotherapeutics in inhibiting tumor growth [11], and participated in the CIPN [12,13]. Chemotherapeutic drugs alter the gut microbiota, leading to acute dysbiosis and modifying physiological and psychological functions. Restoration of the gut microbiota (i.e., using probiotics) may counteract the psycho-physiological impairment occurring in young survivors upon chemotherapy, eventually improving the symptoms and health [14,15].

Notably, Shen and colleagues [16] reported that the pretreatment with antibiotics for three weeks (twice a day) decreased the bacterial load in mouse feces. Moreover, oxaliplatin-induced mechanical hyperalgesia was decreased in germ-free animals and in animals pretreated with antibiotics. Finally, upon oxaliplatin, the infiltration of macrophages and cytokines (IL6 and tumor necrosis factor-α) in the DRGs of antibiotics-treated animals were reduced compared control group [16]. Another study reported that PTX-induced neuropathic pain could be prevented by the probiotic DSF, a high concentration probiotic formulation (450 billion bacteria per sachet) [17]. Thus, this evidence suggested that probiotics may be used as adjuvant therapy for counteracting CIPN. Rousseaux described the first evidence for a link between specific bacteria and the endocannabinoid and opioid systems [18], showing that oral administration of specific Lactobacillus strains modulated the expression of cannabinoid and µ-opioid receptors in gut epithelial cells and reduced abdominal pain in rats. Moreover, opioid receptors are highly expressed within the digestive tract and influence gut motility and gut microbiota composition [19].

Cannabinoids and opioids are known to induce analgesia. Additionally, these receptors and their endogenous ligands have comparable cellular and anatomical distribution through sensory nuclei and associated pathways (e.g., dorsal spinal cord), and their activation induces pain decrease [20]. It is well documented that the two systems are in strict communication. Concurrent activation of cannabinoid and opioid receptors can produce synergistic analgesic effects [21,22] even if they work through separate (although possibly interrelated) mechanisms [23,24,25]. It has been reported that the endogenous opioid system may be implicated in cannabinoid antinociception, and recent works indicated a role of the endogenous cannabinoid system in opioid antinociception [23,26,27].

Natural products are receiving increasing consideration from the research community due to their phytochemical, biological, and pharmacological properties [28,29,30,31]. Probiotic formulations may diminish inflammation through cytokine production [32] and reduce oxidative stress by reducing reactive oxygen species [33]. It has been reported that probiotic formulations can regulate the immune system, reducing the inflammatory factors, in particular, reducing the proliferation of the T-Cells, increasing anti-inflammatory cytokines (IL-10, TGF-β) and decreasing the pro-inflammatory cytokines (including interleukins, TNF-α IFN-γ), nuclear factor kappa-light-chain-enhancer of activated B cells (NF-κB) and further intracellular signaling mediators [34,35]. Consequently, it has been indicated that some probiotic formulations may be useful in the management of inflammatory pain symptoms. [36,37]. It has been demonstrated that the original probiotic formulation DSF can counteract PTX-induced neuropathic pain in vitro [17]. A novel probiotic formulation SLAB51 (high concentrated probiotic formulation sold as Agimixx^®^, Ormendes, Jouxtens-Mézery, Switzerland) was investigated for the neuroprotective effects in neurodegenerative disorders [38,39].

Based on this evidence, in this work, we tested the hypothesis that the probiotic formulation SLAB51 could counteract PTX-induced neuropathic pain in vivo. For this purpose, we tested the probiotics in mice treated with PTX evaluating nociceptive behavior and analyzing the inflammatory and the biomolecular pathway involved in CIPN and the gut status. Moreover, we assessed the role of two main pain receptors, CB1 and opioids (µ and K), in probiotic-induced anti-hyperalgesic and antiallodynic activities.

## 2. Materials and Methods

### 2.1. Animals

Behavioral tests were performed on male CD1 mice (25–30 g, Charles Rivers, Calco (Lecco), Italy). They were housed in the animal care facility at the Department of Pharmacy of the University of Naples Federico II, Italy. Animals were housed in a room with controlled temperature (22 ± 1 °C), humidity (60 ± 10%), and light (12 h per day); food and water were available *ad libitum*. All behavioral tests were performed between 09:00 and 14:00 h by researchers blinded to the treatment, and the animals were used only once. After completion of experiments, animals were sacrificed.

### 2.2. Experimental Groups and Procedures

Ten animals per group (for four experimental groups) were used. A total of 40 animals were used.

1. Sham Mice treated with Vehicle (*n* = 10),
2. Sham Mice treated with probiotics (*n* = 10),
3. Mice treated with Paclitaxel + Vehicle (*n* = 10),
4. Mice treated with Paclitaxel + probiotics (*n* = 10)

**Figure 1:**
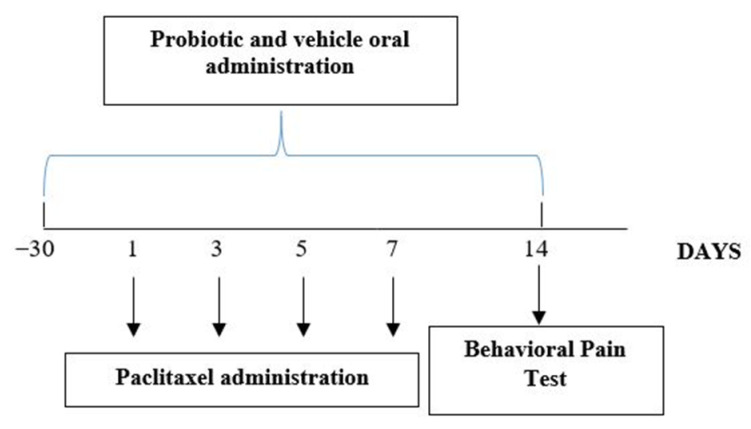
Experimental design showing the time points of probiotic and paclitaxel administration and behavioral tests.

Seven days after the last PTX administration, mice were subjected to behavioral pain tests and then sacrificed, tissue collected for ex vivo experiments (Figure 1).

### 2.3. Drug Treatment

PTX (Tocris, Bristol, UK) was dissolved in a solution made up of 50% Cremophor EL and 50% absolute ethanol and stored at −20 °C, for a maximum of 14 days, and then diluted in normal saline (NaCl 0.9%) to obtain a final concentration of 8 mg/mL just before administration. SLAB51 formulation was provided by Ormendes SA (Switzerland). SLAB51 was freshly prepared by dissolving one sachet (1.5 g/200 billion of bacteria) in 10 mL of drinking water as previously described [39]. SLAB51 and vehicle were given orally for 44 days (as shown in Figure 1) dissolved daily in drinking water.2.4. Chemotherapy-Induced Peripheral Neuropathy

The protocol used was performed according to Toma and coworkers [40]. The animals received PTX 8 mg/kg intraperitoneally (i.p.) for four alternate days (days 1, 3, 5, and 7, as shown in Figure 1); behavioral pain tests were performed on day 14, following PTX injection. Sham mice received an equal volume of vehicle (Cremophor EL: Ethanol, 1:1, diluted in sterile 0.9% NaCl solution to a final concentration of 33.3% Cremophor EL: Ethanol) i.p.

### 2.4. Behavioral Pain Tests

#### 2.4.1. Mechanical Allodynia (Von Frey Test)

To evaluate the development of mechanical allodynia or alterations in sensation or sensitivity to tactile stimulation was measured using the Dynamic Plantar Aesthesiometer (DPA, Ugo Basile, Comerio, Italy). Animals were placed in a chamber with a mesh metal floor covered by a plastic dome that enables the animals to walk freely but not to jump. The mechanical stimulus (paw withdrawal threshold, PWT) was then delivered in the mid-plantar skin of the hind paw. The cut-off is fixed at 5 g, while the increasing force rate (ramp duration) is set at 20 s. The DPA automatically records the force at which the foot is withdrawn. The test was performed 2 h after the last oral administration, on both paws on day 14 after paclitaxel administration.

#### 2.4.2. Mechanical Hyperalgesia (Randall-Sellitto Test)

Paw withdrawal threshold (PWT) to mechanical pressure was measured with a Randall-Selitto Analgesy-Meter (Ugo Basile, Italy). The test consists of the application of arising mechanical force, in which the tip of the device was applied onto the medial portion of the plantar surfaces of hind paws until a withdrawal response resulted. The Randall-Selitto Analgesy-Meter records the force at which the foot is withdrawn. The test was performed 2 h after the last oral administration, on both paws on day 14 after PTX administration. The cut-off was fixed at 130 g.

### 2.5. Ex Vivo Experiments

After in vivo experiments, mice were anesthetized with isoflurane (4%) and then euthanized. Spinal cord, colon, and serum were collected for further analyses.

#### 2.5.1. Serum TNF-α, IL-1β, and IL-6 Determination

On day 14, mice were euthanized, and blood was collected by cardiac puncture. Serum was obtained by centrifugation at 1500× *g* at 4 °C for 15 min and stored at −70 °C. TNF-α, IL-1β, and IL-6 levels (ng/mL) were measured by ELISA kits for mice from BD Pharmingen, according to the manufacturer’s protocols.

#### 2.5.2. Western Blot Analysis

Colon and spinal cord tissues were removed and homogenized on ice-cold lysis buffer with protease and phosphatase inhibitors. After 30 min on ice, lysates were centrifugated at 16,000× *g* for 30 min at 4 °C, and the supernatants were stored at −80 °C until use. Protein concentration was evaluated by the Bio-Rad protein assay (Bio-Rad, Hercules, CA, USA) using bovine serum albumin (Sigma–Aldrich, Milan, Italy) as standard. An equal amount of protein was run on sodium dodecyl sulfate-polyacrylamide minigels and transferred onto nitrocellulose membranes (Protran Schleicher & Schuell Bioscience, Dassel, Germany) or polyvinylidene fluoride. Aspecific binding sites were blocked with 3% nonfat dried milk (Bio-Rad, Hercules, CA, USA) for 45 min at room temperature, and then incubated overnight at 4 °C with the following primary antibodies in the same blocking solution: anti-iNOS (1:1000, BD Bioscience, Buccinasco, Italy); anti-COX-2 (1:1000; BD Bioscience, Buccinasco, Italy); anti-CB1 (1:1000, Novus Biologicals, Cambridge, UK), anti-µ receptor (1:1000, Novus Biologicals, Cambridge, UK), anti-ᴋ receptor (1:1000, Novus Biologicals, Cambridge, UK), anti-ZO-1 (1:1000, Invitrogen, Milan, Italy); anti-occludin (1:500, Santa Cruz Biotechnology, Santa Cruz, CA, USA), rabbit anti-acetylated α tubulin (1:1000, Cell Signaling, Danvers, MA, USA); rabbit anti-pFAK (1:500, Cell Signaling, Danvers, MA, USA); rabbit anti-pSTAT3 (1:1000, Cell Signaling, Danvers, MA, USA); rabbit anti-pJAK2 (1:200, Santa Cruz, USA); rabbit anti-PPARγ (1:1000, Abcam, Cambridge, MA, UK); and HRP-conjugated Actin 1:10,000 (Cell Signaling, Danvers, MA, USA). After extensive washes, the membranes were incubated with the appropriate secondary antibody (Jackson Immuno Research, West Grove, PA, USA) for 1 h at RT. After other washes, the antibody-reactive bands were revealed by chemiluminescence. Signals were visualized using ImageQuant 400 (GE Healthcare, Milan, Italy), equipped with Quantity One Software 4.6.3 (Bio-Rad, Hercules, CA, USA). Anti-β-actin was used as housekeeping protein.

#### 2.5.3. Immunohistochemistry Analyses

Animals were sacrificed, and biopsies (3 mm) were taken from the right hind paw pad. Samples were immediately placed in Zamboni’s fixative, where they were left at 4 °C. As a decalcifying protocol, the paws were placed in 10% EDTA (pH 7.4) as previously described [41]. Samples were then placed in 30% sucrose for at least 24 h and used for immunohistochemistry analysis. Tissues were included in Optimal Cutting Temperature compound (OCT, Thermo Fisher Scientific, Waltham, MA, USA) and sliced into 10 μm thick serial coronal sections by cryostat (Thermo Scientific, USA). Briefly, sections were blocked with phosphate buffer saline (PBS) containing 4% BSA for 2 h at RT and then incubated with the following primary antibodies overnight at 4 °C: IL-8 (mouse, 1:100, Santa Cruz, USA), PGP9.5 (rabbit, 1:1000, Abcam, USA), and collagen IV (goat, 1:25, Invitrogen, USA). After different washes with PBS, sections were incubated with secondary antibodies for 2.30 h at RT, donkey AlexaFluor 488 anti-rabbit, donkey AlexaFluor 633 and goat anti-mouse Alexafluor 546 (1:2000; Life Technologies, USA). Finally, coverslips were mounted with Vectashield with DAPI (Vector Laboratories, Burlingame, CA, USA) and then observed at a Leica TCS SP5 confocal microscope (Leica, Wetzlar, Germany).

#### 2.5.4. Statistical Analyses

Data and statistical analysis in this study conform with the recommendations on experimental design and research in pharmacology [42]. Statistical analyses were performed using Prism 9 Graphpad software (GraphPad Software Inc., San Diego, CA, USA). All data are presented as mean ± SEM. In the Randall-Selitto test, data are presented as paw withdrawal threshold (g). Mechanical allodynia data are presented as mechanical withdrawal threshold (g). For all experimental data, two-way repeated measurements ANOVA followed by post hoc Bonferroni’s multiple comparison test determined the significance of differences between groups. A *p* < 0.05 was considered statistically significant for all tests.

## 3. Results

### 3.1. Probiotic Treatment Reduces Colon Tissue Damage

**Figure 2:**
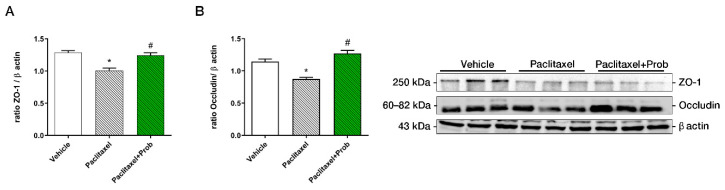
Western blotting analyses for ZO-1 (**A**) and occludin (**B**) in the distal colon. *, *p* < 0.05; vs. vehicle; #, *p* < 0.05 vs. paclitaxel (*n* = 3). A representative figure for each protein is shown.

To evaluate barrier integrity, the expression in the distal colon of two tight junction proteins, occludin and zonulin-1 (ZO-1) was performed. Paclitaxel-treated mice showed occludin and ZO-1 expression lower than those of the vehicle group (Figure 2A,B, * *p* < 0.05 vs. vehicle). Preventive probiotic treatment significantly restored occludin and ZO-1 expression throughout the colonic mucosa (Figure 2A,B ^#^ *p* < 0.05 vs. paclitaxel). These data suggest that the probiotic could prevent the damage due to chemotherapy preserving the intestinal barrier integrity and the expression of the tight-junction proteins.

### 3.2. Effect of Probiotic on Paclitaxel-Induced Mechanical Allodynia and Hyperalgesia

**Figure 3:**
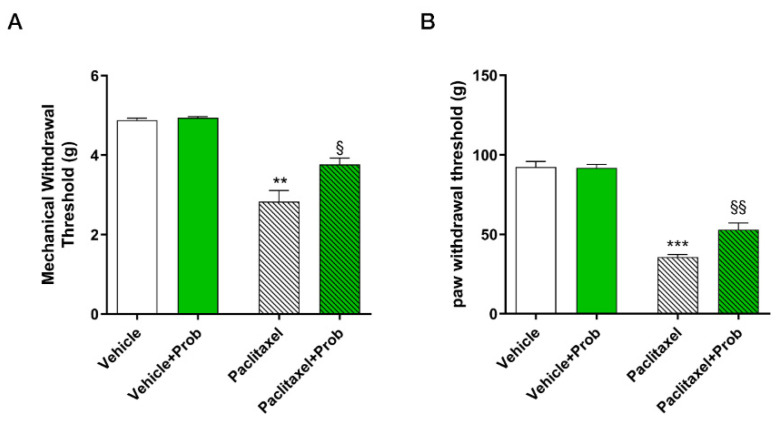
Behavioral tests: (**A**) Von Frey test to evaluate mechanical allodynia (**B**) Randall-Selitto test to evaluate mechanical hyperalgesia. ***, *p* < 0.0001; **, *p* < 0.005; vs. vehicle; §§, *p* < 0.005; §, *p* < 0.05 vs. paclitaxel (*n* = 10).

As illustrated in Figure 3A, no difference was observed between vehicle-sham (white bar) and probiotic-sham (green bar) mice. As expected, repeated PTX treatment-induced neuropathic pain; in fact, paclitaxel treated animals showed a significant reduction in mechanical allodynia if compared to the vehicle group (** *p* < 0.01; Figure 3A). Probiotic treatment in the paclitaxel mice group was able to increase the mechanical withdrawal threshold in a significant manner (§ *p* < 0.05) with respect to paclitaxel treated animals (Figure 3A). Data on mechanical hyperalgesia induced by neuropathy are in agreement with mechanical allodynia data. No significant difference was observed between vehicle-sham (white bar) and probiotic-sham (green bar) mice groups (Figure 3B). Paclitaxel treated mice showed significant hyperalgesia compared to sham vehicle-treated mice (*** *p* < 0.0001, Figure 3B), while the probiotic treatment in paclitaxel-treated mice showed a significant anti-hyperalgesic effect with respect to paclitaxel treated animals (§§ *p* < 0.005; Figure 3B).

### 3.3. Probiotic Treatment Modulates CB-1, µ, and ĸ Receptors and Pro-Inflammatory Proteins Expression in the Spinal Cord

**Figure 4:**
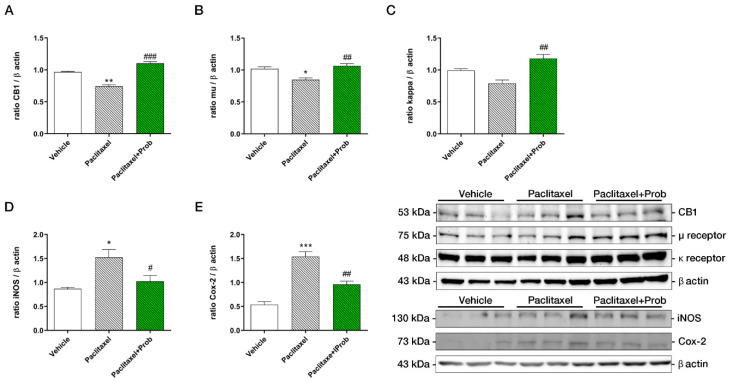
Western blotting analyses for CB1 (**A**), µ (**B**) and kappa (**C**) receptors, iNOS (**D**), and Cox-2 (**E**) proteins in spinal cords. ***, *p* < 0.0001; **, *p* < 0.005; #, *, *p* < 0.05 vs. vehicle; ###, *p* < 0.0001, ##, *p* < 0.005; #, *p* < 0.05 vs. paclitaxel (*n* = 3). A representative figure for each protein is shown. Western blotting analyses for CB1, µ and kappa receptors, iNOS, and Cox-2 proteins in spinal cords. ***, *p* < 0.0001; **, *p* < 0.005; vs. vehicle; ##, *p* < 0.005; #, *p* < 0.05 vs. paclitaxel (*n* = 3). A representative figure for each protein is shown.

Western blot analysis confirmed the involvement of paclitaxel in the development and maintenance of pain hypersensitivity by mechanical stimuli since it was able to reduce CB-1, µ, and kappa receptors expression in the spinal cord if compared to the vehicle group (Figure 4A–C, * *p* < 0.05 vs. vehicle). Moreover, the results also confirmed the anti-nociceptive role of probiotic treatment: in fact, its administration increased significantly cannabinoid and opioid receptor expression (Figure 4A–C, ^##^ *p* < 0.005 vs. paclitaxel). Additionally, we assessed the inflammatory state by evaluating two critical enzymes, cyclooxygenase-2 (COX-2) and inducible nitric oxide synthase (iNOS), at the spinal cord level. The protein levels of iNOS and COX-2 were higher in paclitaxel-treated animals’ spinal cord with respect to the vehicle group (Figure 4D,E, * *p* < 0.05 and *** *p* < 0.0005, respectively). Preventive oral probiotic treatment significantly reduced the expression of the two pro-inflammatory markers with respect to the paclitaxel group (Figure 4D,E, ^#^ *p* < 0.05 vs. paclitaxel), underlining the anti-inflammatory effect of the probiotic treatment.

**Figure 5:**
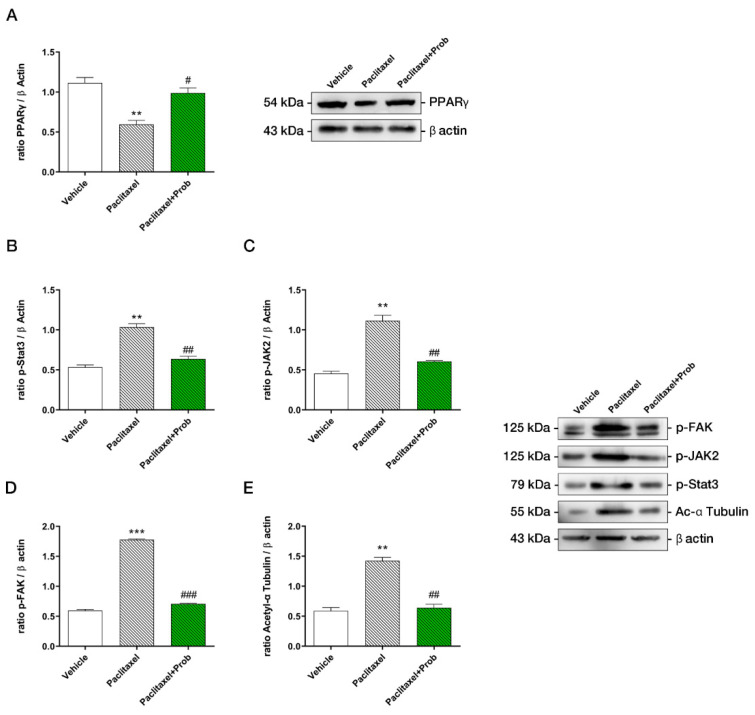
Western blotting analyses for PPARγ (**A**), p-STAT3 (**B**), p-JAK2 (**C**), p-FAK (**D**), acetyl α-tubulin (**E**) in spinal cords. ***, *p* < 0.0001; **, *p* < 0.005; vs. vehicle; ###, *p* < 0.0005; ##, *p* < 0.005; #, *p* < 0.05 vs. paclitaxel (*n* = 3). A representative figure for each protein is shown.

Finally, to better evaluate the inflammatory state following chemotherapy, we assessed peroxisome proliferator-activated receptor gamma (PPARγ) spinal cord expression due to its well-known role in CIPN and control of inflammation [43]. Interestingly, in the paclitaxel-treated group, a significant reduction of the transcription factor was apparent. In parallel, the probiotics increased PPARγ protein levels (Figure 5A), thus suggesting that its activation could be responsible for the reduced neuroinflammation.

### 3.4. Probiotic Treatment Counteracts the Increase of Neuropathic Pain Proteins

In agreement with previous findings [17,44], PTX significantly increased proteins involved in establishing neuropathic pain in the spinal cord (Figure 5B,E), such as p-Stat3, p-Jak2, p-FAK, acetylated α-tubulin, all involved in microtubule stabilization and long-lasting pain sensation. Probiotic treatment restored the control levels of all protein assayed, confirming in vivo the results already obtained in vitro on sensitive neurons [17].

### 3.5. Effects of the Probiotics on Inflammatory Markers in Serum

**Figure 6:**
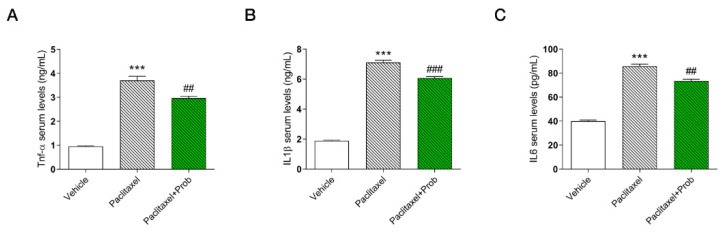
Pro-inflammatory Tnf-α (**A**) and IL1β (**B**) and IL6 (**C**) concentrations in serum analyzed by ELISA. ***, *p* < 0.0001; vs. vehicle; ###, *p* < 0.0005; ##, *p* < 0.005 vs. paclitaxel (*n* = 3).

Based on these results, we also evaluated the systemic anti-inflammatory effect of probiotic analyzing pro-inflammatory TNF-α and IL1β and IL6 concentrations in serum. Results clearly showed a significant increase of all pro-inflammatory mediators in the paclitaxel group with respect to the vehicle group (Figure 6A–C, *** *p* < 0.0001 vs. vehicle). Probiotic treatment counteracted this effect; in fact, all pro-inflammatory mediators analyzed, Tnf-α and IL1β and IL6, were significantly decreased (Figure 6A–C, ##, *p* < 0.005; ###, *p* < 0.0001; ##, *p* < 0.005 vs. paclitaxel respectively). These results confirmed the probiotic formulation’s anti-inflammatory effects as already observed in the analyses of PPARγ protein levels.

### 3.6. Probiotic Treatment Prevents the Paclitaxel-Induced Loss of Intra-Epidermal Fiber (IENFs) in the Paw

**Figure 7:**
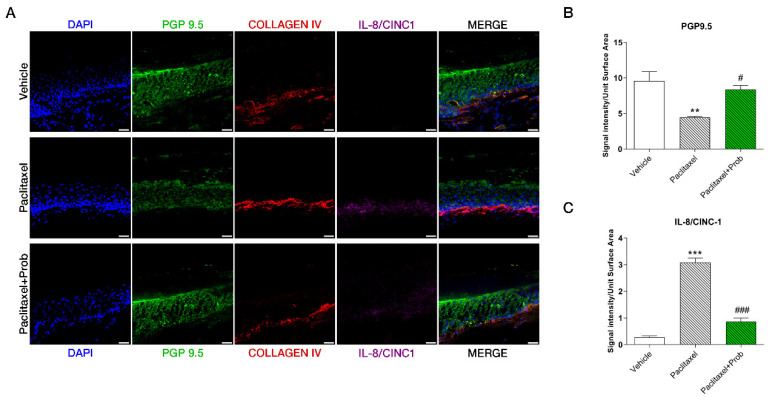
(**A**) Immunofluorescence analyses at confocal microscopy for PGP 9.5 (green), collagen IV (red), IL-8/CINC1 (purple), DAPI (blue) in the paw, scale bar: 25 µm. A representative figure for each marker is shown. Signal intensity analyses for PGP 9.5 (**B**) and IL8 (**C**). **, *p* < 0.005; ***, *p* < 0.0001; vs. vehicle; ###, *p* < 0.0001; #, *p* < 0.05 vs. paclitaxel (*n* = 3).

IENFs loss plays a significant role in neuropathies in response to chemotherapeutic agents, including PTX [45,46]. The immunofluorescence levels of PGP9.5 (a marker of IENFs) and collagen IV in the paw revealed impairment in IENFs, extending from derma into the epidermis in paclitaxel-treated animals in comparison to control mice (Figure 7A,B). Probiotic treatment significantly counteracted this event, decreasing epidermal innervation loss (Figure 7A,B). Moreover, immunofluorescence analysis for murine interleukin 8 (IL-8/CINC1) showed that PTX sharply increased this marker in the dermis (Figure 7A,C). Probiotic treatment strongly attenuated this event, triggering a protective effect on nerve fibers (Figure 7C). Altogether, these results reveal that PTX leads to a substantial reduction in IENFs, which was counteracted by the probiotic administration.

## 4. Discussion and Conclusions

CIPN is one of the most common dose-limiting side effects of chemotherapy [4]. Indeed, PTX is a high-efficiency anticancer medicine, but neuropathic pain represents this treatment’s major side-effect. It has been reported that the effects of paclitaxel are dose-dependent, with the higher doses triggering axonal degeneration and lower doses triggering hypersensitivity pain, including allodynia and hyperalgesia [47]. Neurotoxicity induced by PTX is ascribed to altered microtubule structure, which induces enhanced microtubule stability by increasing acetylated α-tubulin triggering neuropathic pain, but it has been recently reported that PTX also causes inflammation in DRG neurons in culture [48,49]. Although the mechanism by which PTX injures peripheral sensory fibers is not identified, it has been indicated that PTX impairs axoplasmic transport [50], leads to neuronal mitochondria dysfunction [51], and supports epithelial damage leading to axonal degeneration [52].

The analgesic drugs used to relieve pain symptoms in CIPN are few, and they do not efficiently relieve symptoms. It is worth noting that, currently, no recommended options for effective prevention of CIPN are available.

In this study, we evaluated the effect of SLAB51, a mix of probiotics, in PTX-induced neuropathic pain and inflammation state with the aim to verify if a preventive probiotic treatment might improve neuropathic conditions and thus it may constitute a valid approach to improve drug treatments for neuropathic pain. The results reported here describe for the first time the efficacy of the probiotic formulation SLAB51 in in vivo models of PTX-induced peripheral neuropathy.

Dysbiosis-induced systemic inflammation can independently and bidirectionally initiate or aggravate stress circuits, particularly the hypothalamic-pituitary-adrenal (HPA)-axis, via the vagus nerve [53,54]. Chemotherapeutics alter the gut microbiota in both pediatric and adult patients. Cancer treatments, especially chemotherapy, can drive microbial gut dysbiosis [55], alter immune, metabolic and HPA-axis function [56], but also induce psychological and cognitive impairment [57,58]. Upon chemotherapeutics, a substantial reduction of Actinobacteria and Firmicutes and an increase of Proteobacteria were detected paralleled by a decreased capacity for nucleotide, energy, and vitamin metabolism, indicating that chemotherapeutics lead to numerous imbalances in the gut microbiome [14].

Gut dysbiosis is hypothesized to be linked with many disorders, both serious peripheric consequences (i.e., altered intestinal barrier and visceral pain) [11,59] and also at the central level (including depression, epilepsy, and pain) [60]. Thus, it is relevant to maintain and restore a “healthy” microbiome to manage or prevent chronic conditions. The healthy gut microbiota communicates a particular function in host nutrient metabolism, xenobiotic and drug metabolism, gut barrier maintenance, immunomodulation, and protection against pathogens [61,62]. In particular, the maintenance of gut barrier integrity is fundamental for the microbiome equilibrium [63,64]. In our experimental conditions, the chemotherapeutic altered the barrier integrity, evaluated by the level of tight-junction protein expressions ZO-1 and occludin. Results clearly show that preventive probiotic supplement was able to restore the gut barrier integrity. Several peripheral and central diseases’ etiology is also due to deregulation gut microbiota after damage in epithelial barrier function [65,66]. An altered gut microbiota profile is related to metabolic impairment such as obesity [67] and insulin resistance [68] and altered intestinal permeability [69]. Moreover, increased intestinal permeability is a causal factor of systemic inflammation and could induce persistent pain. Many articles show how abnormal gut microbiota composition and alteration of intestinal permeability can contribute to the development of several pain conditions such as irritable bowel syndrome, neuropathic and inflammatory pain [16,70,71]. In 2007, Rousseaux showed the correlation between microbiota and endocannabinoids and opioids receptors, suggesting that intestinal flora’s modulation may be a promising, safe and relatively low-cost novel treatment for abdominal pain [18].

The endocannabinoids and opioids receptors have a crucial part in mediating pain-induced analgesia. The endocannabinoid system exerts is crucial for energy balance, pain response, processing of central and peripheral pain signals, learning and memory, reward, and emotions [72]. On the other hand, also opioid receptors represent a useful tool for pain management. Over the last decade, numerous groups investigated the cannabinoid/opioid crosstalk in antinociception, drug reinforcement, and anxiety [73,74].

In our experimental conditions, we showed two important findings by prolonged probiotic treatment (i) it reduced allodynia and hyperalgesia associated by PTX administration and (ii) it was able to modulate opioid and cannabinoid spinal receptors.

Preclinical studies have determined the involvement of gut microbiota in several animal pain models, such as in visceral pain [75] in inflammation [76] pain and chronic neuropathic pain [11,71]. In agreement with these findings, our results show that probiotic formulation can reduce hyperalgesic and allodynic state due to PTX administration, and these effects are probably mediated by cannabinergic and opioidergic systems. In fact, following prolonged SLAB51 treatment CB1 MU and Kappa spinal receptors were significantly increased respect to PXT-mice. In our opinion, combined activation of cannabinoid and opioid system may cause analgesic effects that are greater than those generated by the activation of separate classes of receptors.

Moreover, the inflammatory process triggered by chemotherapeutics has been proposed as a potential starter of the nociceptive pain in CIPN [77,78], and proinflammatory chemokine release upon chemotherapeutics has been indicated as one of the leading events affecting the neuro-immune communication. Pro-inflammatory cytokines can trigger neural damage by inflammatory process and a direct activity on neurons and glial cells [79,80,81,82].

Notably, the inflammatory mediators, including iNOS and COX-2 are strongly reduced at the spinal cord level upon the treatment, suggesting a cumulative effect in activating signaling, decreasing pro-inflammatory pathways. In line with these results, PPARγ, known to trigger anti-inflammatory actions and down-regulate iNOS and COX2 levels, appears downregulated by paclitaxel and restored to control levels probiotics.

Furthermore, the chemotherapeutics led to a substantial reduction in IENFs, which was considerably counteracted by the probiotics. Even if the mechanism of epidermal denervation is still unclear, some studies indicated that it is associated with PTX-neurological toxicity [83,84], and is related with the increased release of pro-inflammatory cytokines [77,84]. Notably, we observed that IENF loss corresponds to a local increase of serum pro-inflammatory cytokine release induced by PTX and that the probiotic treatment significantly reduced this phenomenon, suggesting its critical protecting role in nerve fibers.

The results obtained in this in vivo study supported our previous in vitro observations on probiotics effects in counteracting PTX-induced neuropathic pain [17].

In summary, it is possible to claim that probiotics were able to perform different actions on PTX side effects: they could decrease inflammation and fiber loss in the paws. This event is probably a consequence of a general decrease in circulating cytokines and inflammatory proteins at spinal cord levels and the induction of anti-inflammatory protein PPARγ. The results regarding PPARγ confirmed our previous results on another CNS model in which we observed a nuclear translocation of this receptor upon probiotics treatment, thus suggesting its activation [39]. These effects were accompanied by positive modulation of cannabinoid and opioid receptors, thus indicating a combined probiotic treatment action.

Overall, it is possible to postulate that the probiotic was able to prevent PTX-induced neuropathic pain, resulting in the reduction in gut permeability, and in nerve fiber damage, decreasing the inflammation, thus suggesting that the use of this specific probiotic formulation (SLAB51) may represent a valid adjuvant agent to PTX, useful and not toxic for long lasting treatments. On this basis, it is possible to propose probiotics during chemotherapy cycles to decrease the side effects of these drugs.

## Data Availability

The datasets are available from the corresponding authors upon reasonable request.
